# Does Ammonia Released from Protein-Based Attractants Modulate the Capture of *Anastrepha obliqua* (Diptera: Tephritidae)?

**DOI:** 10.3390/insects12020156

**Published:** 2021-02-12

**Authors:** Rodrigo Lasa, Trevor Williams

**Affiliations:** Red de Manejo Biorracional de Plagas y Vectores, Instituto de Ecología AC (INECOL), Xalapa, Veracruz 91073, Mexico

**Keywords:** hydrolyzed proteins, torula yeast, ammonia solution, traps, pH, West Indian fruit fly

## Abstract

**Simple Summary:**

The West Indian fruit fly, *Anastrepha obliqua* (family Tephritidae), is an important pest of mango and guava in the Neotropical region. Previous studies have indicated that ammonia is involved in fruit fly attraction to sources of food, particularly if protein is present. In laboratory experiments, flies were attracted to ammonia solutions of increasing concentration. In contrast, fly captures by different protein-based attractants were not related to the quantity of ammonia released by the attractant. Flies also responded differently to yeast suspensions of different alkalinity that released different amounts of ammonia. In field experiments, flies were strongly attracted to yeast in ammonia solutions after 24 h, but this effect did not persist when measured over a 7-day period. We conclude that *A. obliqua* flies are attracted to ammonia solutions in the absence of other stimuli, but attraction to protein-based attractants or alkaline yeast suspensions is not correlated with the quantity of ammonia released by these substances. Ammonia is an important component in fruit fly attraction, which also seems to depend on the presence of other compounds derived from protein food sources in different stages of decomposition.

**Abstract:**

Tephritid fly responses to food-based attractants involve a complex range of food-derived semiochemicals, including ammonia. We performed laboratory and field experiments to compare the attraction of *Anastrepha obliqua* (Macquart) to ammonia with the attraction to commercial food attractants and torula yeast at a range of pHs. A positive correlation was established between the concentration of ammonia in solution (1.5–150 mM ammonium solution) and gaseous ammonia released by bottle-type traps. This resulted in an asymptotic response in captures of *A. obliqua* flies in traps that released 99–295 µg ammonia/h. Pairwise comparisons in laboratory cages revealed that traps baited with 150 mM ammonia solution captured similar numbers of *A. obliqua* as traps baited with Biolure 2C, CeraTrap, and hydrolyzed protein products (Captor, Winner, and Flyral) plus borax, despite the low quantities of ammonia (11–56 µg/h) released from these attractants. Subsequent choice experiment captures in traps containing ammonia solution were similar or higher than those of commercial attractants, with the exception of Winner + borax, but were not correlated with the ammonia released from attractants. Captures of flies in traps containing ammonia solution were increased by the addition of 1% torula yeast or torula yeast alkalized with sodium hydroxide or borax despite differences in the quantities of ammonia released. Fly captures generally increased with increasing alkalization of torula yeast (pH 7.5–9.5). In the field, torula yeast in ammonia solution captured similar numbers of *A. obliqua* flies as Captor + borax when traps were evaluated after 24 h but not after a 7-day trapping period. Traps baited with ammonia solution or Winner + borax were significantly less attractive than Captor + borax in both field experiments. We conclude that *A. obliqua* flies are attracted to ammonia solutions of increasing concentration, up to 150 mM, in the absence of other stimuli, whereas attraction to commercial attractants or alkalized torula yeast is not correlated with the release of ammonia.

## 1. Introduction

Tephritid fruit flies are one of the most economically important agricultural pests worldwide [1]. The West Indian fruit fly, *Anastrepha obliqua* (Macquart; Diptera: Tephritidae), is distributed from central Mexico to eastern Brazil [2]. It is an important pest of mango, *Mangifera indica* L., tropical plum, *Spondias* spp., and occasionally guava, *Psidium guajaba* L. [2]. This fly is considered a potential threat to other tropical and subtropical regions of the world [3]. A better understanding of the pest’s responses to food lures is needed to improve detection methods in quarantine regulation areas and as one component of the integrated pest management tools available for this fly.

Proteinaceous food lures are currently the most effective attractants used for trapping and controlling *Anastrepha*, an economically important genus for which effective host-based lures have yet to be developed [4]. Like most other pestiferous *Anastrepha* species, *A. obliqua* is currently monitored using liquid lures based on hydrolyzed protein, torula yeast plus borax, or synthetic attractants that release ammonia [5,6]. Dietary proteins are key nutrients required for female ovarian development and to increase egg production or to improve male sexual performance and promote longevity [7,8]. The need for protein and its scarcity in agroecosystems are likely to motivate the attraction of adult flies to volatile substances emitted from proteins, such as ammonia [4,9,10]. The earliest observations of attraction to ammonia in fruit flies occurred during early tests on lures for *Anastrepha ludens* (Loew) [11]. Later, borax (sodium tetraborate) was included in protein-based attractants because it preserves flies caught in traps [12]. Borax also increases the pH of protein lures, which improves their attractiveness to several species of tephritids [13,14]. The increase in pH results in an increased release of ammonia, likely due to the reaction of ammonium and hydroxide ions to produce ammonia gas and water [9,15,16,17].

Although it is clear that ammonia is emitted from protein-baited traps, the influence of attractant composition and pH on ammonia release and adult tephritid responses has not been subjected to systematic examination. This may be due to the somewhat complex procedures required to quantify ammonia emissions. As a result, most studies have been performed using ammonia solutions of known concentration and dry protein-based attractants [9,10], patches containing ammonium salts [18,19,20], or mixtures of food-based baits with ammonium bicarbonate or ammonium acetate [15,17,21].

The present study was performed under laboratory conditions to evaluate the attraction of *A. obliqua* to a range of concentrations of ammonia in aqueous solution (ammonium hydroxide). Fly responses to traps baited with ammonia solution were systematically compared with several commercial food attractants, including hydrolyzed proteins and ammonium acetate plus putrescine patches, and with alkalized torula yeast at a range of pHs. Field studies were then performed to compare fly captures in traps baited with two standard hydrolyzed proteins or ammonia solution or a mixture of ammonia solution + torula yeast.

## 2. Material and Methods

### 2.1. Laboratory Rearing of Anastrepha obliqua

A colony of *A. obliqua* was started at the Instituto de Ecología AC, Xalapa, Veracruz State, Mexico, using pupae that emerged from naturally infested wild tropical plum, *Spondias mombim* L., collected from Tuzamapan, Veracruz State (19°25′4″ N; 96°52′18″ W, 957 m altitude) in October 2019. Pupae were removed from trays at 3–4 day intervals and placed in groups of 200 in transparent plastic cups (200 mL) containing 10 g of vermiculite moistened with 0.3% (wt/vol) sodium benzoate solution. Cups were covered with a nylon mesh lid and placed in 60 × 60 × 90 cm mesh-covered cages for adult eclosion. Adults of both sexes were kept together in cages and maintained in a climatically controlled room at 24 ± 1 °C, with 60 ± 10% relative humidity (RH) and a 12:12 h (L:D) photoperiod. Groups of 30–40 mated females and 5–10 males of 15–35 days old were transferred to 30 × 30 × 30 cm acrylic cages and were allowed to oviposit in insecticide-free green mangoes var. Manila. Pupae were recovered from these fruits and used for the following generation of insect production. Flies of both sexes, for use in experiments, were collected from adult emergence cages at age 7–11 days and were assumed to be sexually mature but unmated. All adult flies had continuous ad libitum access to hydrolyzed yeast, sugar, and water prior to experiments.

### 2.2. Traps, Attractants, and Experiment Sites

Two types of bottle-shaped traps were used in experiments: (i) a colorless PET (polyethylene terephthalate) plastic bottle (500 mL) was adapted as a trap by making three 11-mm diam. circular holes spaced 5 cm apart at 2/3 height of the trap; (ii) a commercial MS2^®^ trap of 600 mL capacity (Fitozoosanitaria SA de CV, Texcoco, Mexico), consisting of a yellow base cup with a transparent top that had three 11-mm diam. circular holes spaced 5 cm apart. The colorless bottle trap was used for laboratory studies involving colorless attractants. The MS2 trap, with its opaque yellow base, was used to compare attractants with different colors in the laboratory (to avoid fly responses to particular colors) and for both field experiments.

Several lures, commonly used in Mexico to trap this pest, were tested, including three commercial chemical hydrolyzed proteins: (i) Captor 300 (Química Lucava, Celaya, Guanajuato, Mexico), (ii) Winner 360 (International Química de Cobre SA de CV, Mexico City, Mexico), and (iii) Flyral (Bioibérica, Barcelona, Spain). A 10 mL volume of each chemically hydrolyzed protein product was mixed with 5 g of borax (J.T. Baker, Mexico City, Mexico) and 235 mL of water, as is indicated in the Mexican federal government guidelines for fruit fly lures [5]. An enzymatic hydrolyzed protein of animal origin, CeraTrap^®^ (Bioibérica, Barcelona, Spain) was also evaluated without dilution or the inclusion of borax, following the manufacturer’s recommendations. The dry lure Biolure^®^ 2C (Suterra Inc., Bend, OR, USA) was also included in experiments. This attractant comprises separate sachets of ammonium acetate and putrescine, both of which have an adhesive strip for attachment to the trap. Biolure^®^ 2C-baited traps also contained 250 mL of water as the drowning solution, with 12 µL of Tween 80 (Golden Bell, Materiales y Abastos Especializados SA de CV, Mexico City, Mexico) as a wetting agent. Other experiments used inactive powdered torula yeast (Lake States type BD75, Lallemand Inc., Montreal, Canada), a common ingredient in tephritid meridic diets.

### 2.3. Determination of pH and Quantification of Released Ammonia

The pH of lures and experimental solutions was measured using a digital pH meter (Okpow, China) 10 min after the solution was prepared. The accuracy of meter readings was checked against buffers of known pH (7.0, 10.0) prior to each measurement. All solutions and protein preparations were prepared using bottled drinking water (Xallapan, Grupo Doso SA de CV, Xalapa, Mexico) with a pH of 7.1–7.3 in order to reduce possible variations in the pH of each attractant [13].

The release of gaseous ammonia from ammonia solutions or protein hydrolysate mixtures was determined using an apparatus previously designed for this purpose [9]. Collection of ammonia was performed using a 5-L opaque glass jar (255 mm height, 140 mm diam.) in which a plastic bottle trap containing 250 mL of each experimental solution or attractant was placed (Figure S1). The glass jar was sealed with a plastic lid that was perforated with two holes through which PTFE (polytetrafluoroethylene) tubes were passed. Air was injected into the jar with the aid of an aquarium air pump Elite 800 (RC Hagen UK Ltd., Castleford, UK). Injected air had previous passed through an activated charcoal filter and was regulated using a flowmeter (Cole-Parmer Scientific, Vernon Hills, IL, USA) at a rate of 150 mL/min. Another PTFE tube inserted into the other hole of the lid allowed air and volatiles to exit the jar and was connected to a 55 mL capacity glass tube (25 × 150 mm) containing 10 mL of distilled water to dissolve ammonia. Air was diffused into the water using an aquarium stone (Grupo Acuario de Lomas SA de CV, Mexico City, Mexico) attached to the end of the tube (Figure S1). The glass water trap was sealed with a perforated rubber bung and a glass tube to allow the escape of exhaust gas. Preliminary tests performed by us demonstrated that ≥95% of the ammonia gas was captured in this tube of water, thus obviating the need for a second ammonia trap tube previously used by others [9].

The ammonia that dissolved in the distilled water trap was determined by measuring ammonium ion concentration through its reaction with Nessler reagent, quantified using an ammonia medium-range photometer (Hanna Instruments Inc., Woonsocket, RI, USA). The Nessler reagent (K_2_HgI_4_) reacts with ammonium ions to produce a yellow-colored product, the intensity of which is directly proportional to the quantity of ammonia present. All measurements were performed in a climatically controlled laboratory at 24 ± 1 °C, following the manufacturer’s recommendations. As ammonia release varied widely among experimental preparations, the duration of the capture time ranged from 10 min to 5 h. This procedure resulted in concentrations between 1 and 5 mg/L of ammonia in the distilled water trap, in line with the accuracy range of the ammonia photometer. The final quantity of ammonia was subsequently adjusted to a standard 1-h capture time and expressed in micrograms of ammonia per hour (µg/h).

### 2.4. Ammonia Released from Traps Baited with Ammonia Solution

Bottle PET traps were prepared, containing 250 mL of one of six different concentrations of ammonia solution (26° Baumé, 28.3–29.4% (wt/vol), 0.89 mg/mL density): 0.003–1% vol/vol, equivalent to 0.5–150 mM of ammonia. Baited traps were prepared 16–20 h before each assay and placed in 5-L opaque glass jars for determination of the rate of ammonia release, as described in the previous section. Five replicate traps were used for each ammonia concentration. The relationship between the ammonia concentration in solution and the rate of gaseous ammonia release was determined by simple linear regression using the R-based package Jamovi v.1.2.27.0 [22], which was also used for all subsequent analyses.

### 2.5. Attraction to Different Concentrations of Ammonia

A laboratory choice experiment was performed to evaluate the capture of *A. obliqua* flies in traps containing one of six concentrations between 0.5 and 150 mM ammonia solution. A 250 mL volume of each solution was deposited in a 500 mL PET bottle trap, which was placed in an acrylic cage (30 × 30 × 30 cm). A total of six traps was placed in a hexagonal arrangement in a randomized sequence with a 15-cm distance between adjacent traps. Ammonia solutions were prepared 16–20 h prior to each experiment. All solutions included 12 µL of Tween 80 to improve the likelihood of flies drowning in the traps. A group of 30 female and 30 male nonstarved flies of 7–11 days old was released inside the cage in the morning (10–11 a.m.). Trapped flies were collected from each trap 23 h later, counted, and sorted by sex. Untrapped flies were discarded. All tests were performed under the same laboratory conditions used to rear the insect colony. Traps were then washed, loaded with new ammonia solutions, and subsequently rotated clockwise by one position for each new replicate (six replicates per cage performed over a 6-day period). In this way, each concentration was tested once at each trap position within each cage. The experiment was performed in five different cages, giving a total of 30 replicates for each concentration. Mean trapped flies were √(x + 0.5) transformed to stabilize the variance and subjected to a two-way ANOVA for ammonium concentration and fly sex. Mean comparisons were performed by the Tukey HSD test.

### 2.6. Attraction to Ammonia or Commercial Attractants under Choice Conditions

Two experiments were performed to compare fly attraction to different commercial lures with their attraction to an ammonia solution. In the first experiment, pairwise comparisons were performed for the 150 mM ammonia solution, identified as the most attractive ammonia concentration in the previous experiment, and each of five different commercial attractants; (i) Captor + 2% borax, (ii) Winner + 2% borax, (iii) Flyral + 2% borax, (iv) CeraTrap, and (v) Biolure 2C. For this, two MS2 traps, loaded with either 250 mL ammonium hydroxide or 250 mL commercial lure, were placed at opposite sides of acrylic cages (30 × 30 × 30 cm) and initially assigned to random positions. The position of each trap was subsequently switched for each new replicate. Attractants were prepared one day before use. In the case of ammonia solution, 12 µL of Tween 80 was included as a wetting agent. For Biolure, a dry lure, 250 mL of water with 12 µL of Tween 80 was used as the drowning solution, and patches were stuck inside the upper section of the bottle trap. As in the previous experiment, a group of 30 female and 30 male nonstarved flies of 7–11 days old was released inside each cage. All tests were performed under the same conditions used to rear the laboratory insect colony. Flies captured in traps were collected 23 h later, counted, and sorted by sex. Uncaptured flies inside the cage were discarded. Each test was performed in six independent cages, and traps were evaluated at both positions within each cage, giving twelve replicates in total. Mean numbers of trapped males and females and percentage of females captured were compared by paired *t*-tests. The ammonia released by 250 mL of each commercial attractant was measured from a sample of five replicate traps using the opaque jar and the ammonium photometer method. Means of ammonia releases from the 150 mM ammonia solution and commercial attractants were compared by paired Welch’s *t*-test to account for unequal variances.

In the second experiment, the 150 mM ammonia solution and the five commercial attractants were evaluated simultaneously in a six-way choice experiment. Traps were hung randomly in a hexagonal arrangement in 60 × 60 × 90 cm mesh-covered cages with a 30 cm distance between adjacent traps. All other procedures were identical to those of the pairwise choice experiment but performed at 26 ± 1 °C, 65 ± 10% relative humidity (RH), and a 12:12 h (L:D) photoperiod with a light intensity of 1000–1500 lux, measured at the top of the cage. After each 23 h capture period, flies were counted and sorted by sex; traps were washed, baited with new attractants, and rotated clockwise by one position for each new replicate (six replicates per cage, one attractant at each position). The experiment was performed simultaneously in four independent cages, giving a total of 24 replicates. Mean numbers of trapped flies were √(x + 0.5) transformed to stabilize variance and subjected to two-way ANOVA for attractant and fly sex, followed by a Tukey HSD test.

### 2.7. Attraction to Torula Yeast in Ammonia Solution

Four experiments were performed to examine fly responses to traps baited with totula yeast mixed with ammonia solution. First, attraction to 150 mM ammonia solution was compared with a solution comprising 1% (wt/vol) torula yeast + 150 mM ammonia solution (pH 10.6) using MS2 traps loaded with 250 mL of each attractant. This experiment was performed in acrylic cages (30 × 30 × 30 cm), as described in the previous pairwise experiments. The experiment was performed in four independent cages under laboratory rearing conditions, and each trap position was switched once to give a total of eight replicates.

Second, as the addition of torula yeast to 150 mM ammonia solution significantly increased fly captures, an additional experiment compared fly attraction to 1% torula yeast + 150 mM ammonia solution against Winner + 2% borax. This is because the mixture of Winner + borax was identified as the most attractive hydrolyzed protein in the previous six-way choice experiment.

Finally, two similar experiments were performed to compare fly attraction to 1% (wt/vol) torula yeast + 150 mM ammonia solution against 1% (wt/vol) torula yeast alkalized with sodium hydroxide to a similar pH (pH 10.6) or with 2% (wt/vol) borax (pH 9.3).

In all cases, mean trapped males and females and the percentage of captured females were compared between treatments by paired *t*-tests or a Wilcoxon rank test in one case of non-normally distributed data. The ammonia released by each baited trap was captured by individually placing five replicate traps in opaque glass jars and measured as described previously. Means of ammonia releases from traps were compared by paired Welch’s *t*-test for unequal variances.

### 2.8. Attraction to Alkalized Torula Yeast

The attraction of *A. obliqua* flies to an aqueous suspension of 1% (wt/vol) torula yeast (pH 6.8) was compared with a 1% torula yeast suspension alkalized to pH 7.5, pH 8.5, and pH 9.5 by the addition of 0.1 N sodium hydroxide solution. MS2 traps loaded with 250 mL of each attractant were evaluated in a four-way choice experiment in 60 × 60 × 90 cm mesh cages. Traps were randomly hung in each corner at a distance of ~45 cm between adjacent traps, following the methodology used in previous experiments. Attractants were prepared 16–20 h before the experiment. All yeast suspensions included 12 µL of Tween 80 to reduce the surface tension of the liquids and improve the likelihood of flies drowning. As before, a group of 30 female and 30 male nonstarved flies of 7–11 days old was released inside each cage. Traps were baited with new attractants and subsequently rotated clockwise by one position for each new replicate (four replicates, one at each cage position). Treatments were evaluated simultaneously in four different cages, giving a total of 16 replicates. The ammonia released from yeast at different pHs was measured from five replicate traps, as described previously. Sodium hydroxide solution alone released no ammonia. Mean numbers of flies captured, percentage of captured females, and the rate of ammonia release by alkalized torula yeast at different pHs were subjected to one-way ANOVA, followed by a Tukey HSD test for mean comparisons.

### 2.9. Attraction to Ammonia and Commercial Protein Attractants under Field Conditions

Two field experiments were performed during May–July 2020 in an area of 1 km^2^ of mango orchards (19°19′40″ N, 96°45′25″ W, 340 m altitude) surrounded by tropical plum near the village of Jalcomulco in central Veracruz, Mexico. During the experimental period, this region was characterized by a mean temperature of 26.5 °C (range 20–33 °C) and daily rainfall, with a mean precipitation of ~160 mm/month. Different attractants were evaluated based on the results of the laboratory studies. In the first experiment, the experimental orchard was divided into four blocks of ~0.5 ha each. MS2 traps were loaded with 250 mL of one of four different attractants: (i) 150 mM ammonia solution, (ii) 1% torula yeast + 150 mM ammonia solution, (iii) Captor + 2% (wt/vol) borax, and (iv) Winner + 2% borax. Four traps, one of each treatment, were placed on mango trees within each block at the height of 2–3 m above the ground. Traps on different trees were separated by 10–12 m. Traps were emptied at 24 h intervals and flies were collected, placed in 70% ethanol, and transported to the laboratory. Each trap was then washed, filled with a new attractant, and rotated one position within the block for the following 24 h period. The experiment was performed over eight consecutive days so that each trap was placed at each position within the block on two occasions.

In the second experiment, traps were distributed among five blocks (~0.5 ha), with one trap per treatment in each block. The distance between traps on different mango trees was, again, 10–12 m. In this case, traps were checked at intervals of 7 days for a period of four consecutive weeks. In this way, each trap was monitored for one week at each of the four positions within each block. Attractants were renewed after each weekly collection of trapped flies.

In the laboratory, captured *Anastrepha* flies were counted and identified to species and sex. The percentage of females of *A. obliqua* was calculated for those traps that captured at least one fly of this species. The mean numbers of trapped *A. obliqua* flies were used to fit GLMs with a Poisson distribution with overdispersion, followed by Bonferroni mean comparisons. Percentages of captured females in field experiments were not normally distributed and were subjected to a nonparametric Kruskal Wallis test. During the second field experiment, four traps per treatment were selected at random, and the attractants were collected at weekly inspection and taken to the laboratory for ammonia release measurements. The quantities of ammonia released from traps following the 7-day period in the field were subjected to ANOVA followed by Tukey HSD mean comparisons.

For clarity, all the experimental comparisons performed under laboratory and field conditions are listed in order in Appendix A.

## 3. Results

### 3.1. Ammonia Released from Traps Baited with Ammonia Solution

**Figure 1:**
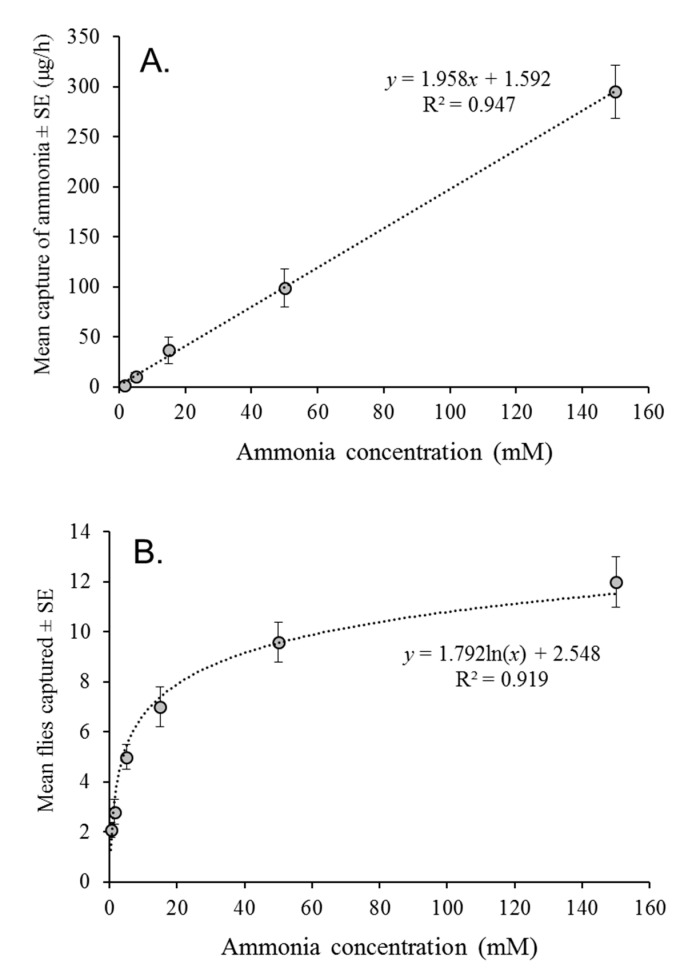
(**A**) Linear regression of ammonia concentration in solution and the quantity of gaseous ammonia released by traps over a one-hour period. (**B**) Logarithmic (ln) regression of mean numbers of flies caught in traps baited with different concentrations of ammonia solution.

A linear regression equation was found to closely predict ammonia release from ammonia solutions in traps (F = 414.0, df = 1, 23, *p* < 0.001; R^2^ = 0.947; Figure 1A). The concentration of 0.5 mM ammonia was not included in the regression as the quantity of ammonia released was below the limits of detection after 5 h of sample collection. The mean (±SE) emission of ammonia varied from 1.2 ± 0.2 µg/h from the 1.5 mM ammonia solution to 295.1 ± 26.5 µg/h from the 150 mM ammonia solution (Figure 1A).

### 3.2. Attraction to Different Ammonia Concentrations

The mean total number of *A. obliqua* flies captured in traps was positively correlated with ln (ammonia concentration; F = 52.23, df = 5, 300, *p* < 0.001; R^2^ = 0.919), with approximately two flies/trap at the concentration of 0.5 and 1.5 mM, increasing to 12 flies/trap at the highest concentration of 150 mM (Figure 1B). Captures did not differ significantly with fly sex (F = 0.0311, df = 1, 300, *p* = 0.860), with similar mean percentages of females captured across all ammonia concentrations (range: 46.8% to 54.8%).

### 3.3. Attraction to Ammonia or Commercial Attractants under Choice Conditions

Pairwise comparisons of the 150 mM ammonia solution with five different commercial attractants revealed no significant differences in the mean capture of flies (Table 1 and Table S1). The percentage of females captured was also similar for all comparisons, except for a significantly higher percentage of females trapped in the 150 mM ammonia solution than with Winner + borax (Table 1). However, the pH of the attractants varied between 6.9 and 9.0, compared to pH 11.3 for the ammonia solution. The quantity of ammonia released from commercial attractants varied from 11.5 ± 3.5 µg/h for CeraTrap to a maximum of 56.1 ± 21.2 µg/h for Biolure 2C, but all were significantly lower than the ammonia release from the ammonia solution (295.1 µg/h; Table 1 and Table S1).

**Figure 2:**
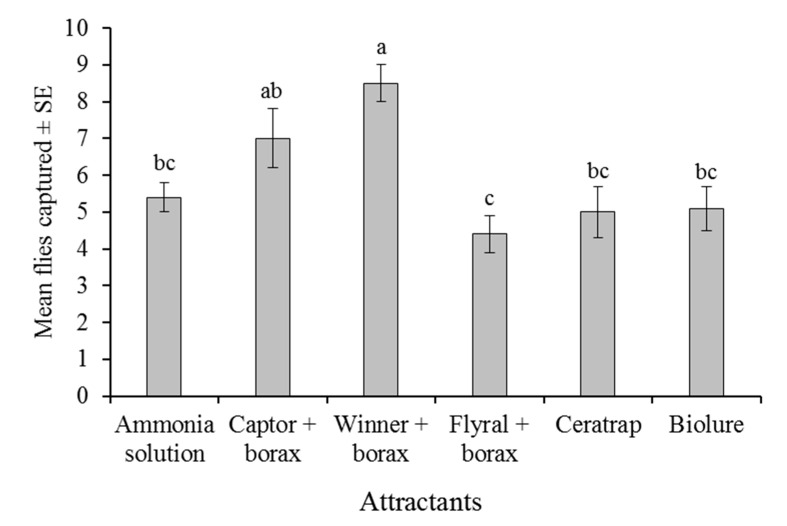
Mean numbers of *A. obliqua* flies captured in traps baited with the 150 mM ammonia solution or five different commercial attractants under a six-way laboratory choice experiment. Columns headed by the same letter do not differ significantly (ANOVA, Tukey, *p* > 0.05).

In a six-way choice experiment, with all commercial attractants and the 150 mM ammonia solution, the mean number of trapped *A. obliqua* flies differed significantly among attractants (F = 7.982, df = 5, 240, *p* < 0.001; Figure 2). The highest mean captures were observed in the traps baited with Winner + borax or Captor + borax and the lowest in the Flyral + borax-baited traps. Ammonia solution and the other attractants had intermediate numbers of captures (Figure 2).

The mean percentage of females captured varied between 39.5 ± 4.9% for CeraTrap and 51.0 ± 3.8% for the 150 mM ammonia solution but did not vary significantly among attractants (F = 3.819, df = 1, 240, *p* = 0.052; data not shown in Figure 2). There was no significant correlation between the number of flies captured and the quantity of gaseous ammonia released by the attractants (determined previously in Table 1; Spearman’s rho = 0.4857, *p* = 0.50).

### 3.4. Attraction to Torula Yeast in Ammonia Solution

The addition of torula yeast to ammonia solution (pH 10.6) resulted in a significant increase in the numbers of flies captured; this combination captured three-fold more flies than Winner + borax (Table 2 and Table S2). Interestingly, yeast adjusted to a high pH by the addition of sodium hydroxide (pH 10.6) or borax (pH 9.3) captured similar numbers of flies as the mixture of yeast and ammonia solution.

Ammonia solution alone and Winner + borax both captured significantly higher percentages of female flies than the mixture of yeast + ammonia solution. In contrast, alkalized yeast captured a similar fraction of females as the mixture of yeast + ammonia solution (Table 2). The addition of yeast to ammonia solution resulted in a marked decrease in the release of ammonia gas from baited traps, but the yeast + ammonia solution suspension consistently released a higher quantity of ammonia than the other combinations tested. The alkalization of torula yeast by the addition of sodium hydroxide or borax resulted in very low levels (1.2–1.9 µg/h) of ammonia emission (Table 2 and Table S2). There was no significant correlation between the mean numbers of flies captured and the quantities of ammonia released from traps (Spearman’s rho = 0.0595, *p* > 0.50).

### 3.5. Attraction to Alkalized Torula Yeast at Different pHs

**Figure 3:**
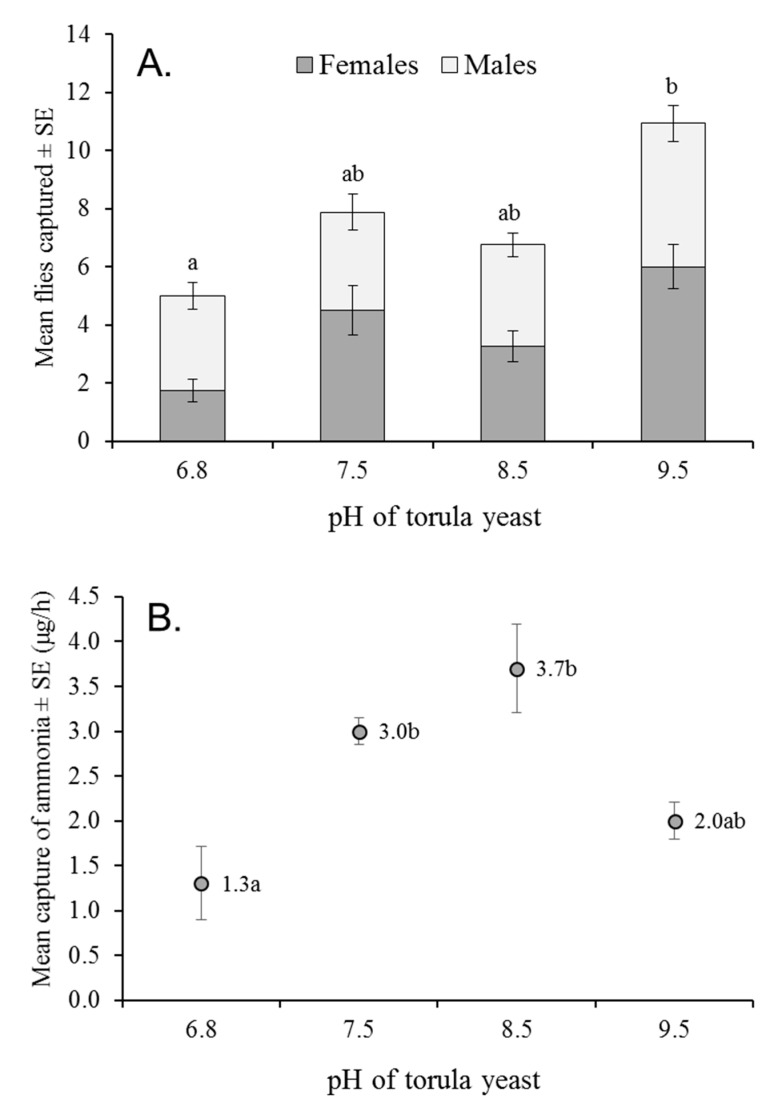
(**A**) Mean numbers of *A. obliqua* males and females captured in traps baited with 1% torula yeast (pH 6.8) or 1% torula yeast alkalized to different pHs. (**B**) Mean quantities of ammonia released by untreated torula yeast or alkalized torula yeast at different pHs. Total flies captured (males + females) in (**A**) and pH values in (**B**) labeled with the same letter do not differ significantly (ANOVA, Tukey, *p* > 0.05).

The mean number of flies caught in traps baited with alkalized torula yeast increased significantly with increasing pH, from 5.0 ± 0.8 flies/trap at pH 6.8 (unaltered pH of yeast) up to 10.9 ± 1.3 flies/trap at pH 9.5 (F = 5.51, df = 3, 60, *p* = 0.002; Figure 3A). The mean percentage of females captured did not differ at any alkalized pH (48–57%, depending on pH). However, a significantly lower percentage of females (30.0 ± 5.2%) than males were captured in the unaltered yeast baited traps (pH 6.8; F = 6.948, df = 3, 46, *p* < 0.001; Figure 3A). The mean release of ammonia from traps baited with alkalized torula yeast also differed significantly with pH, with the highest quantities released at pH 7.5 and pH 8.5, the lowest release at pH 6.8, and an intermediate quantity released at pH 9.5 (F = 9.66, df = 3, 12, *p* = 0.002; Figure 3B).

### 3.6. Attraction to Ammonia Mixtures and Commercial Protein Solutions under Field Conditions

**Figure 4:**
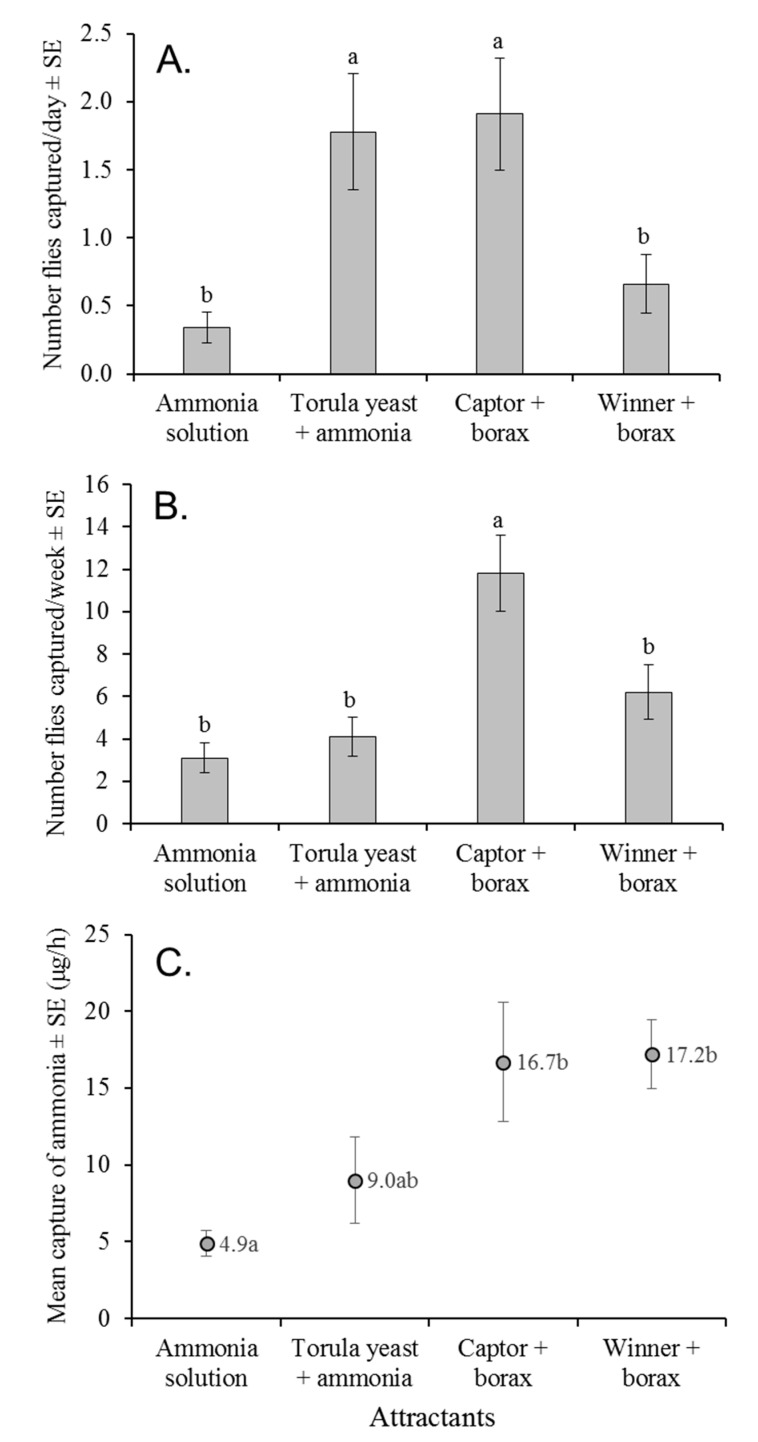
Mean numbers of *A. obliqua* flies captured in traps baited with 150 mM ammonia solution or different attractants under field conditions. (**A**) In the first experiment, traps were sampled at 24 h intervals or (**B**) at weekly intervals in the second field experiment. (**C**) Samples taken from traps after 7 days released varying quantities of ammonia. Columns (**A**,**B**) or points (**C**) labeled with identical letters do not differ significantly ((**A**,**B**) Bonferroni, (**C**) Tukey, *p* > 0.05).

A total of 160 *Anastrepha* spp. flies were collected in the first field experiment, in which traps were inspected after 24 h. Of these, 150 flies were *A. obliqua* (89 females and 61 males), and 10 individuals were *A. serpentina* (4 females and 6 males). Only *A. obliqua* flies were included in the analysis. The mean number of flies trapped per day differed significantly among attractants (GLM: χ^2^ = 23.085, df = 3, *p* < 0.001). The highest captures were registered in traps baited with Captor + borax and torula yeast + ammonia solution, with significantly fewer captures in traps baited with Winner + borax or 150 mM ammonia solution alone (Figure 4A). The mean percentage of females captured varied between 43.8% and 62.9% but did not vary significantly among attractants (Kruskal Wallis: χ^2^ = 3.07, df = 3, *p* = 0.381).

In the second field experiment, a total of 624 *Anastrepha* spp. flies were trapped, of which 501 flies were *A. obliqua* (308 females and 193 males) and 123 were *A. serpentina* (66 females and 57 males). Only *A. obliqua* flies were included in the analysis. The mean number of flies per trap per week varied significantly among attractants (GLM: χ^2^ = 42.43, df = 3, *p* < 0.001), with significantly higher numbers captured in traps baited with Captor + borax compared to any of the other attractants (Figure 4B). The mean percentage of females captured varied between 51.6% and 67.0% but did not differ significantly among attractants (Kruskal Wallis: χ^2^ = 1.30, df = 3, *p* = 0.729). Samples collected from traps after 7 days revealed that the torula yeast + ammonia solution had decomposed and emitted a putrefied odor, which likely influenced the low fly captures in this treatment. The mean ammonia release by traps also differed significantly in samples collected after 7 days in the field (F = 5.00, df = 3, 12, *p* = 0.018). Captor + borax and Winner + borax released similar mean quantities of ammonia (Figure 4C), both of which were significantly higher than the 150 mM ammonia solution alone, which had apparently lost most of the ammonia. The mixture of yeast + ammonia solution released an intermediate quantity of ammonia.

## 4. Discussion

Natural and synthetic food attractants represent the largest, most diverse, and, likely, the most complex area of tephritid semiochemical research [23]. This is due to the variable responses of flies to the different food types and the diversity of tephritid life histories and host plants and their interaction with other endogenous biotic factors such as age, sex, and nutritional status, among others [4,24]. Ammonia is considered one of the most important components that modulate tephritid responses to food-based attractants [9,10,15,17]. Tephritid species vary in their responses to sources of ammonia, which appear to be capable of both additive and synergistic effects if other food-based substances are present, such as grape juice or protein-rich waste from the brewing industry at different stages of decomposition [21].

A positive correlation was initially established between the concentration of ammonia solution (ammonium hydroxide) in bottle-type traps and the quantity of gaseous ammonia released. We quantified ammonia gas using the water trap method [9] and a spectrophotometer kit that uses the Nessler reagent to produce a quantifiable colorimetric reaction. This procedure is simple, rapid, and highly reproducible, as long as the sample is present in cation-free (distilled) water, such as the water used in the present study [25]. Previous efforts to determine ammonia emission from fly attractants have employed the colorimetric phenol–hypochlorite method [9,10,26] and several more complex techniques, such as an ion-selective electrochemical probe [18], solid-phase microextraction [20], and Fourier transform infrared spectroscopy (FTIR) [19,27].

The first laboratory experiment revealed a clear asymptotic response of nonstarved *A. obliqua* flies to traps containing increasing concentrations of ammonia in aqueous solution. The highest captures were observed at the highest concentrations tested (50 and 150 mM ammonia solution), which released between 99 and 295 µg/h of gaseous ammonia (Figure 1A,B). Despite differences in fly species, trapping methodology, nutritional status, and ammonia measurement method, the attraction of *A. obliqua* to ammonia in the present study was similar to the range of ammonia concentrations previously reported to be attractive to other tephritids. For *Ceratitis capitata* (Wiedemann), adult flies were initially reported to respond negatively to ammonia concentrations above 8–17.5 µg/h [9], but a subsequent study determined that concentrations of 13.5–20.5 µg/h of ammonia (released from ~100 mM ammonia solution) were the most attractive for *C. capitata* flies [10]. Traps releasing ~100 µg/h also caught a high number of *C. capitata* flies [10]. Similarly, for *Bactrocera tryoni*, attraction was observed to ammonium bicarbonate solutions that released 3.5–212 µg/h of ammonia, whereas emissions above 283 µg/h ammonia were repellent (values calculated from data from Bateman and Morton [15].

In the case of *Anastrepha* spp., flight tunnel experiments demonstrated that *Anastrepha suspensa* (Loew) was attracted to ammonia concentrations of up to 3840 µg/h, with the strongest attraction to concentrations in the range 60–480 µg/h [27]. The flight responses of mature female flies to ammonia in tunnel bioassays were higher than those of immature flies, although the electroantenography responses of immature female flies (aged 4–6 days) were higher than those of mature females (aged 8–11 days), given a low concentration ammonia stimulus (24 µg/h) [27]. For *A. ludens*, the mean ammonia released by ammonium acetate patches (that were subsequently included in the commercial attractant Biolure 2C) varied between 45 and 405 µg/h [18], whereas a release of 234–373 µg/h of ammonia was established for mixtures of ammonium carbonate, methylamine, and putrescine targeted at *A. ludens* [20].

As far as we are aware, the present study is the first to relate the capture of tephritid flies to the quantity of ammonia released by traps baited with commercial hydrolyzed protein attractants in mixtures with borax. Traps baited with Captor + borax, Flyral + borax, and CeraTrap released very similar concentrations of ammonia (11–20 µg/h), whereas Winner + borax and Biolure 2C released considerably more ammonia (46–56 µg/h). A six-way laboratory choice experiment indicated that attraction to traps baited with 150 mM ammonia solution alone was similar or better than that of the other commercial attractants, with or without borax. The only exception to this finding was Winner + borax, which captured the highest number of *A. obliqua* in the laboratory (Figure 2). However, the number of flies captured was not correlated with ammonia emissions from attractants.

Although ammonia seems to be a key attraction component, our results agree with previous studies in which ammonia alone did not fully account for the attraction of flies [9,15,16,21,28,29]. This was corroborated in our experiments, in which the capture of flies was significantly improved when torula yeast was added to 150 mM ammonia solution (Table 2). This finding underlines the importance of protein-derived volatiles, in addition to ammonia, in the attraction of flies. Indeed, alkalized torula yeast with sodium hydroxide or borax, which released just 1.2–1.9 µg/h of ammonia, captured similar numbers of flies as torula yeast in ammonia solution that released over 50-fold more ammonia (Table 2).

These results, together with a significant increase in fly captures when torula yeast was alkalized to pH 9.5 (Figure 3A,B), although not correlated with increased ammonia, suggest to us that volatile compounds released from alkalized proteins might interact with low concentrations of ammonia, resulting in mixtures that were highly attractive to fruit flies. Indeed, volatile compounds released from alkalized proteins, such as pyrazines, have been implicated in tephritid attraction, whereas volatiles emitted from acidified proteins elicit no such attraction [16,28].

Flies of both sexes generally showed consistent responses to traps baited with ammonia solution or one of the other attractants tested. The only exceptions to this were traps baited with ammonia solution alone or Winner + borax, both of which captured significantly higher percentages of female flies when tested against the mixture of yeast + ammonia solution. In contrast, torula yeast suspension alone (pH 6.8) captured a reduced fraction of female flies for reasons that are unclear. In a previous study, female-biased captures of *C. capitata* in ammonia solutions were observed in experiments involving protein-deprived flies of 2 days old [9], an age and a nutritional condition that were not comparable with our experiments. Sex-based differences in fly responses to ammonia and protein-derived odors likely reflect nutritional requirements that vary during the pre- and postreproductive phases of the adult fly’s life, especially for females that have to invest energy and protein resources in egg production [24].

Interference between plumes emitted by traps in close proximity has been identified as a factor potentially affecting the total capture of a trap in the presence of other traps [30]. Laboratory cage and olfactometer studies, using baited traps at short distances (15–30 cm), have often been used to detect fly attraction to ammonia sources [9,10], proteins mixed with different quantities of borax [13], varying concentrations of proteins or aged baits [31], synthetic compounds [32], or different trap models containing protein baits [33]. As such, tests performed under laboratory conditions, with short distances between traps, can provide information on fly preferences for particular odors; however, the efficacy of specific attractants requires testing under natural conditions with distances of at least ~10 m between traps to minimize interference among odor plumes from nearby traps [34,35,36].

In the field, torula yeast in ammonia solution captured a similar number of *A. obliqua* flies as Captor + borax when traps were evaluated after 24 h. In contrast, after 7 days, torula yeast in ammonia solution had decomposed and emitted a putrid odor that likely reduced fly captures. Traps baited with 150 mM ammonia solution or Winner + borax were significantly less attractive than Captor + borax in both field experiments, despite the fact that Winner + borax captured higher numbers of flies than ammonia solution in laboratory choice experiments. Winner + borax was less effective at trapping flies in the field than Captor + borax, although both lures released a similar quantity of ammonia (~17 µg/h) after 7 days of deployment in the field. We suggest that flies could be more attracted to ammonia when in close proximity to the source, such as in laboratory cages, but not in the field, where volatiles operate over long distances in a complex, volatile environment. This could explain why CeraTrap, which did not outperform other attractants in our laboratory experiments, has proved more effective than Captor + borax for the trapping of *A. obliqua* in the field [37].

## 5. Conclusions 

Our results show that ammonia is a key component for the attraction of *A. obliqua*. In the absence of other stimuli, flies were attracted to ammonia solutions of increasing concentration, up to 150 mM. However, the release of ammonia from commercial attractants or alkalized torula yeast was not correlated with the numbers of flies captured. Future studies should critically examine the interaction of ammonia and protein-derived semiochemicals in the attraction of tephritid flies, which could contribute to improving food-based attractants for monitoring and control programs targeted at these pests.

## Figures and Tables

**Table 1:** Pairwise comparisons of mean (±SE) captures of *A. obliqua* flies, percentage of females captured, and quantity of ammonia released from individual traps baited with the 150 mM ammonia solution and different commercial attractants.

Treatment	Mean No. Flies Captured ± SE	Percentage of Females ± SE	pH	Ammonia Released (µg/h ± SE)
Ammonia solution	17.5 ± 1.7 a	52.6 ± 3.7 a	11.3	295.1 ± 26.5 a
Captor + borax	20.3 ± 1.7 a	47.7 ± 2.5 a	9.0	20.4 ± 2.7 b
Ammonia solution	12.0 ± 2.3 a	57.4 ± 3.4 a	11.3	295.1 ± 26.5 a
Winner + borax	16.8 ± 1.8 a	50.1 ± 2.4 b	8.5	45.8 ± 3.6 b
Ammonia solution	16.5 ± 1.6 a	48.5 ± 3.2 a	11.3	295.1 ± 26.5 a
Flyral + borax	17.3 ± 1.4 a	46.6 ± 2.3 a	8.7	14.2 ± 1.4 b
Ammonia solution	16.8 ± 2.8 a	53.7 ± 2.4 a	11.3	295.1 ± 26.5 a
CeraTrap	20.5 ± 1.8 a	50.4 ± 1.9 a	6.9	11.5 ± 3.5 b
Ammonia solution	14.3 ± 2.4 a	49.1 ± 2.3 a	11.3	295.1 ± 26.5 a
Biolure 2C	17.5 ± 1.7 a	50.0 ± 2.9 a	n/a	56.1 ± 21.2 b

Means followed by identical letters did not differ significantly for pairwise comparisons between ammonia solution and each protein attractant, paired *t*-test for mean numbers of flies captured and percentage of females (*p* > 0.05), or Welch’s *t*-test for ammonia released from traps (*p* > 0.05). All statistical values are presented in Table S1; n/a = not applicable, as Biolure 2C is a dry lure that has no associated pH. The drowning water in Biolure 2C-baited traps was pH 7.4.

**Table 2:** Paired comparisons of mean (±SE) captures of *A. obliqua* flies, percentage of females captured, and quantities of ammonia released from individual traps baited with the 150 mM ammonia solution alone or in mixtures with 1% torula yeast, Winner + borax, or torula yeast alkalized by the addition of sodium hydroxide or borax.

Treatment	Mean No. Flies Captured ± SE	Percentage of Females ± SE	pH	Ammonia Released (µg/h ± SE)
Yeast + ammonia solution	24.0 ± 1.5 a	43.7 ± 2.1 a	10.6	93.9 ± 27.9 a
Ammonia solution	14.0 ± 1.2 b	56.0 ± 3.6 b	11.0	295.1 ± 26.5 b
Yeast + ammonia solution	20.5 ± 1.7 a	40.2 ± 3.3 a	10.6	93.9 ± 27.9 a
Winner + borax	6.4 ± 1.1 b	54.3 ± 5.5 b	8.5	45.8 ± 3.6 b
Yeast + ammonia solution	26.8 ± 1.8 a	46.4 ± 1.4 a	10.6	93.9 ± 27.9 a
Yeast + sodium hydroxide	22.0 ± 1.8 a	52.5 ± 1.8 a	10.6	1.9 ± 0.4 b
Yeast + ammonia solution	26.4 ± 1.4 a	45.4 ± 1.5 a	10.6	93.9 ± 27.9 a
Yeast + borax	23.6 ± 1.8 a	49.7 ± 2.3 a	9.3	1.2 ± 0.1 b

Means followed by identical letters did not differ significantly for each pairwise comparison performed, paired *t*-test for mean numbers of flies captured and percentage of females (*p* > 0.05), or Welch’s *t*-test for ammonia released from traps (*p* > 0.05). All statistical values are presented in Table S2.

## Data Availability

The data presented in this study are available in the supplementary file Data.xlsx.

## Supplementary Materials

The following are available online at https://www.mdpi.com/2075-4450/12/2/156/s1. Figure S1: Apparatus with water trap, used to capture the ammonia released by baited traps. Table S1: Paired *t*-test comparisons of mean captures of *A. obliqua* flies, percentage of females captured, and Welch’s *t*-test of ammonia released from traps baited with 150 mM ammonia solution and different commercial attractants. Table S2: Paired *t*-test comparisons of mean captures of *A. obliqua* flies, percentage of females captured, and Welch’s *t*-test of ammonia released from traps baited with 150 mM ammonia solution alone or in mixtures with 1% torula yeast, Winner + borax, or alkalized torula yeast with sodium hydroxide or borax.

## Appendix A

List of experiments performed on *Anastrepha obliqua* under laboratory cage and field conditions:

- LABORATORY
  - Estimation of release of ammonia gas from traps containing 1.5–150 mM ammonia solution
  - Six-way choice comparison of captures of *A. obliqua* flies in traps containing six concentrations (0.5–150 mM) of ammonia solution
  - Pairwise comparison of *A. obliqua* captures in traps containing 150 mM ammonia solution or one of six commercial attractants (Captor + borax, Winner + borax, Flyral + borax, CeraTrap, Biolure 2C)
  - Six-way choice comparison of *A. obliqua* captures in traps containing 150 mM ammonia solution or one of six commercial attractants (Captor + borax, Winner + borax, Flyral + borax, CeraTrap, Biolure 2C)
  - Comparison of captures in traps containing 150 mM ammonia or 1% torula yeast + 150 mM ammonia
  - Comparison of captures in traps containing 150 mM ammonia or Winner + borax
  - Comparison of captures in traps containing 150 mM ammonia or 1% torula yeast + sodium hydroxide (pH 10.6)
  - Comparison of captures in traps containing 150 mM ammonia or 1% torula yeast + borax (pH 9.3)
  - Four-way comparison of captures in traps containing 1% torula yeast alone (pH 6.8) or 1% torula yeast + sodium hydroxide (pH 7.5, pH 8.5, pH 9.5)
- FIELD TRIALS
  - Four-way comparison of 24 h captures of *A. obliqua* flies in traps containing 150 mM ammonia, 1% torula yeast + 150 mM ammonia, Captor + borax, and Winner + borax
  - Four-way comparison of 7-day captures of *A. obliqua* flies in traps containing 150 mM ammonia, 1% torula yeast + 150 mM ammonia, Captor + borax, and Winner + borax
