# Supplementary material for: Does Ammonia Released from Protein-Based Attractants Modulate the Capture of Anastrepha obliqua (Diptera: Tephritidae)?
Source: Insects. 2021 Feb 12;12(2):156. doi: 10.3390/insects12020156 (PMC7918865; doi:10.3390/insects12020156)
Supplement: Supplementary file 1 [file insects-12-00156-s001.zip › suppl_files/Supplemental material Fig S1 Table S1 S2.pdf]

Figure S1. Apparatus with water trap used to capture the ammonia released by baited traps.

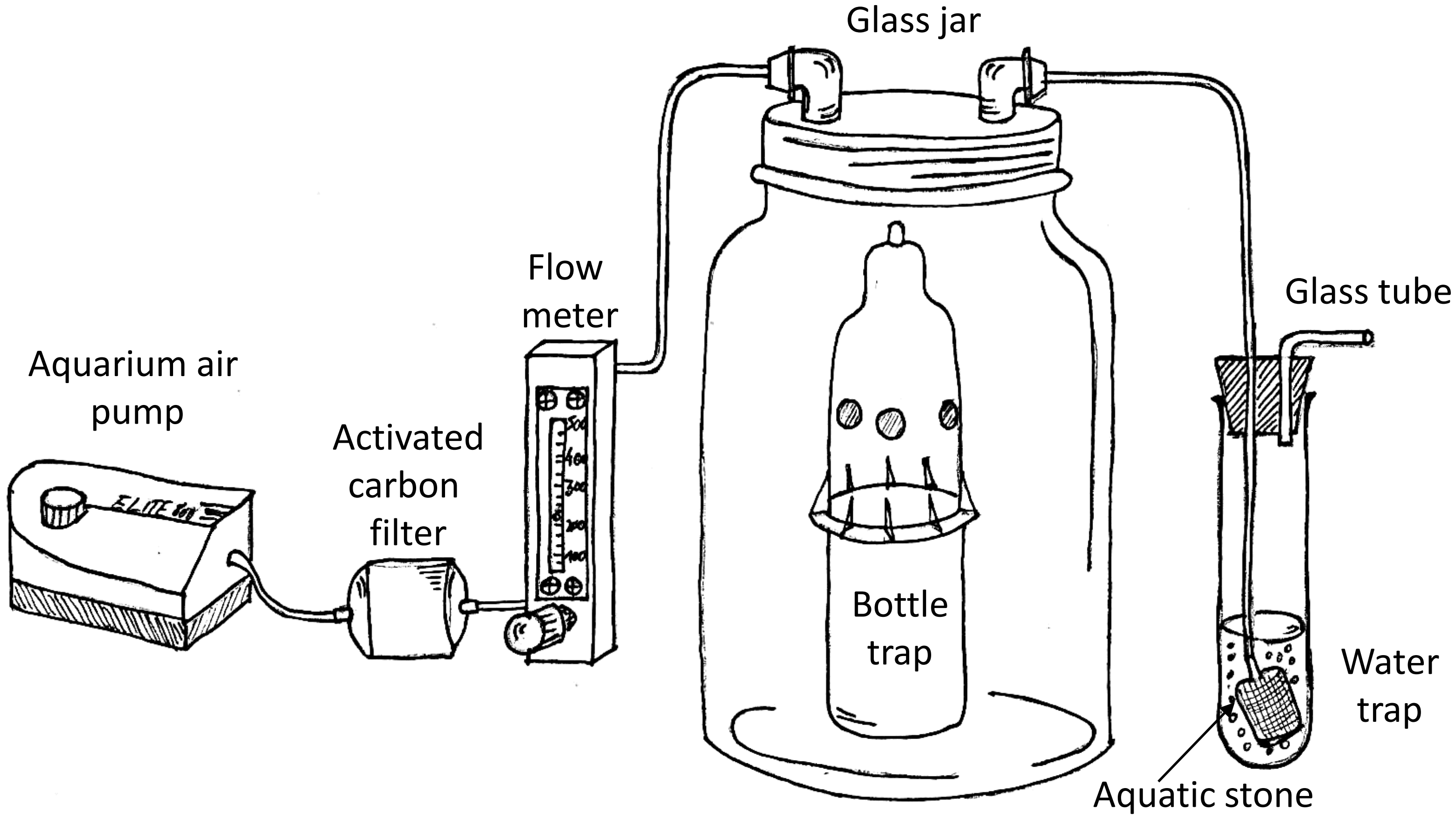

Table S1. Paired t-test comparisons of mean captures of *A. obliqua* flies, percentage of females captured and Welch's t-test of ammonia released from traps baited with 150 mM ammonia solution and different commercial attractants.

Attractant	Mean captures ± SE	Paired t-test	Percentage of females ± SE	Paired t-test	pH	Ammonia captured (µg/h ± SE)	Welch's t-test
Ammonia solution	17.5 ± 1.7	t=0.934, df=11, p=0.370	52.6 ± 3.7	t=0.898, df=11, p=0.388	11.3	295.1 ± 26.5	t=10.30, df=4.06, p<0.001
Captor + borax	20.3 ± 1.7		47.7 ± 2.5		9.0	20.4 ± 2.7	
Ammonia solution	12.0 ± 2.3	t=1.49, df=11, p=0.165	57.4 ± 3.4	t=2.44, df=11, p=0.033	11.3	295.1 ± 26.5	t=9.33, df=4.12, p<0.001
Winner + borax	16.8 ± 1.8		50.1 ± 2.4		8.5	45.8 ± 3.6	
Ammonia solution	16.5 ± 1.6	t=0.275, df=11, p=0.788	48.5 ± 3.2	t=0.463, df=11, p=0.652	11.3	295.1 ± 26.5	t=10.60, df=4.02, p<0.001
Flyral + borax	17.3 ± 1.4		46.6 ± 2.3		8.7	14.2 ± 1.4	
Ammonia solution	16.8 ± 2.8	t=1.29, df=11, p=0.224	53.7 ± 2.4	t=1.05, df=11, p=0.317	11.3	295.1 ± 26.5	t=10.70, df=4.03, p<0.001
Ceratrap	20.5 ± 1.8		50.4 ± 1.9		6.9	11.5 ± 3.5	
Ammonia solution	14.3 ± 2.4	t=0.457, df=11, p=0.656	49.1 ± 2.3	t=0.256, df=11, p=0.803	11.3	295.1 ± 26.5	t=8.49, df=5.00, p<0.001
Biolure 2C	15.4 ± 1.8		50.0 ± 2.9		-	56.1 ± 21.2	

Table S2. Paired t-test comparisons of mean captures of *A. obliqua* flies, percentage of females captured and Welch's t-test of ammonia released from traps baited with 150 mM ammonia solution alone or in mixtures with 1% torula yeast, Winner + borax, or alkalized torula yeast with sodium hydroxide or borax.

**Attractants**	**Mean capture ± SE**	**Paired t-test**	**Percentage females ± SE**	**Paired t-test***	**Ammonia capture (µg/ h ± SE)**	**Welch's t-test**
Yeast + ammonia solution	24.0 ± 1.5	t=4.14, df=7, p=0.004	43.7 ± 2.1	t=2.65, df=7, p=0.035	93.9 ± 27.9 a	t= 5.53, df=7.97, p<0.001
Ammonia solution	14.0 ± 1.2		56.0 ± 3.6		295.1 ± 26.5 b	
Yeast + ammonia solution	20.5 ± 1.7	t=8.180, df=7, p<0.001	40.2 ± 3.3	t=2.58, df=7, p=0.036	93.9 ± 27.9 a	t= 1.91, df=4.13, p=0.126
Winner + borax	6.4 ± 1.1		54.3 ± 5.5		45.8 ± 3.6 a	
Yeast + ammonia solution	26.8 ± 1.8	t=1.15, df=7, p=0.177	46.4 ± 1.4	W=5.0, df=7, p=0.078	93.9 ± 27.9 a	t= 3.69, df=4.00, p=0.006
Yeast + sodium hydroxide	22.0 ± 1.8		52.5 ± 1.8		1.9 ± 0.4 b	
Yeast + ammonia solution	26.4 ± 1.4	t=0.928, df=7, p=0.384	45.4 ± 1.5	t= 1.33, df=7, p=0.224	93.9 ± 27.9 a	t=3.71, df=4.00, p=0.006
Yeast + borax	23.6 ± 1.8		49.7 ± 2.3		1.2 ± 0.1 b	

*In one case a Wilcoxon rank test (W statistic) was used to compared non-normally distributed data
